# Multiple air pollutant exposure is associated with higher risk of all-cause mortality in dialysis patients: a French registry-based nationwide study

**DOI:** 10.3389/fpubh.2024.1390999

**Published:** 2024-07-30

**Authors:** Aghiles Hamroun, Michaël Génin, François Glowacki, Bénédicte Sautenet, Karen Leffondré, Antoine De Courrèges, Luc Dauchet, Victoria Gauthier, Florian Bayer, Mathilde Lassalle, Cécile Couchoud, Philippe Amouyel, Florent Occelli

**Affiliations:** ^1^Service de Santé Publique, Epidémiologie, Economie de la Santé et Prévention, CHU de Lille, Lille, France; ^2^UMR1167 RID-AGE, Institut Pasteur de Lille, INSERM, Université de Lille, CHU Lille, Lille, France; ^3^ULR 2694 - METRICS: Évaluation des Technologies de Santé et des Pratiques Médicales, Université de Lille, CHU Lille, Lille, France; ^4^Service de Néphrologie, CHU de Lille, Lille, France; ^5^Service de Néphrologie-Hypertension Artérielle, Dialyses, Transplantation Rénale, CHRU de Tours, Tours, France; ^6^Department of Nephrology, Université de Tours, Tours, France; ^7^INI-CRCT, Vandœuvre-lès-Nancy, France; ^8^INSERM U1246 SPHERE, Université de Tours-Université de Nantes, Tours, France; ^9^INSERM, Bordeaux Population Health Research Center, Université de Bordeaux, Bordeaux, France; ^10^Coordination Nationale Registre REIN, Agence de la Biomédecine, Saint-Denis, France; ^11^IMT Lille Douai, JUNIA, ULR LGCgE, Laboratoire de Génie Civil et Géo-Environnement, Université de Lille, Université de Artois, Lille, France

**Keywords:** air pollution, environmental exposure, all-cause mortality, dialysis, particulate matter, nitrogen dioxide

## Abstract

**Background:**

Little is known about the effect of combined exposure to different air pollutants on mortality in dialysis patients. This study aimed to investigate the association of multiple exposures to air pollutants with all-cause and cause-specific death in dialysis patients.

**Materials and methods:**

This registry-based nationwide cohort study included 90,373 adult kidney failure patients initiating maintenance dialysis between 2012 and 2020 identified from the French REIN registry. Estimated mean annual municipality levels of PM_2.5_, PM_10_, and NO_2_ between 2009 and 2020 were combined in different composite air pollution scores to estimate each participant’s exposure at the residential place one to 3 years before dialysis initiation. Adjusted cause-specific Cox proportional hazard models were used to estimate hazard ratios (HRs) per interquartile range (IQR) greater air pollution score. Effect measure modification was assessed for age, sex, dialysis care model, and baseline comorbidities.

**Results:**

Higher levels of the main air pollution score were associated with a greater rate of all-cause deaths (HR, 1.082 [95% confidence interval (CI), 1.057–1.104] per IQR increase), regardless of the exposure lag. This association was also confirmed in cause-specific analyses, most markedly for infectious mortality (HR, 1.686 [95% CI, 1.470–1.933]). Sensitivity analyses with alternative composite air pollution scores showed consistent findings. Subgroup analyses revealed a significantly stronger association among women and fewer comorbid patients.

**Discussion:**

Long-term multiple air pollutant exposure is associated with all-cause and cause-specific mortality among patients receiving maintenance dialysis, suggesting that air pollution may be a significant contributor to the increasing trend of CKD-attributable mortality worldwide.

## 1 Introduction

Chronic kidney disease (CKD) is a highly prevalent chronic illness that affects an estimated 10% of the global population, with a significantly higher incidence among older people (1). CKD has been associated with an increased risk of cardiovascular events, hospitalizations, and mortality, especially in patients with kidney failure requiring dialysis (2). Mortality attributed to CKD is steadily increasing worldwide, with an estimated increase of over 30% since 2005, according to the Global Burden of Disease (3). These deaths are primarily of cardiovascular and infectious origin, accounting for up to 70% of the causes of mortality within the dialysis patient population (4).

Recently, air pollution has emerged as a potential contributor to the mortality associated with CKD (5). Air pollution is a complex mixture of gasses and particles of various sizes and compositions, originating from a range of sources such as road traffic, urban heating, industry, or agriculture (6). Since the first description of diesel inhalation-induced kidney damage in mouse models (7), the association between air pollution and kidney health has become a topic of increasing interest, with mounting evidence linking exposure to air pollutants with the incidence of CKD and kidney failure (8, 9). However, there is a paucity of data on the potential impact of air pollutants on mortality in dialysis patients, who are a particularly vulnerable population due to their advanced age and the burden of their comorbidity (10). The few published studies exhibit significant heterogeneity in their design and exposure assessment and are predominantly focusing on all-cause mortality. Moreover, conventional analyses of air pollutants on an individual basis, exclusively reported in the literature on the subject, may not fully capture the complex exposure patterns that occur in real-life settings (11, 12). Composite spatial indices considering multi-contamination have recently been developed and are now recognized as relevant indicators for the global assessment of air quality (13–15).

Based on a nationwide registry and spatial composite air pollution scoring methods, the study’s primary objective is to evaluate the potential impact of multiple exposures to air pollutants on mortality in incident dialysis patients. Secondary objectives include examining the association between multiple exposures to air pollutants and specific causes of mortality, such as cardiovascular and infectious-related deaths.

## 2 Materials and methods

### 2.1 Study setting and population

The STROBE guidelines for cohort studies and a checklist were used in the preparation of this report (Supplementary Table S1). All individuals aged 18 years or older who began maintenance dialysis in France between 1 January 2012 and 31 December 2020 were identified in the REIN database, which serves as the national registry for end-stage kidney disease in France (16). The registry tracks all patients who initiate maintenance kidney replacement therapy in the country and captures patient data at the start of dialysis as well as specific events, such as transplantation and death. Demographic information, including age, sex, and body mass index, as well as data on comorbidities such as cardiovascular diseases, diabetes, chronic respiratory disease, active malignancy, cirrhosis, smoking status, and mobility (classified as walking without help, requiring assistance, or being dependent) are recorded. Emergency dialysis is defined as any first treatment initiated in life-threatening circumstances that require dialysis within 24 h. Biological data such as hemoglobin and serum albumin levels at the start of dialysis are also recorded.

### 2.2 Exposure assessment

#### 2.2.1 Air pollution indicators

Air pollution indicators were obtained from the historical reconstruction of background air pollution in France run by the National Institute for the Industrial Environment and Risks (INERIS). This dataset covers 21 years of air pollution concentrations and air quality indicators in France. Using a kriging method that combines background measurements of air quality and modeling with the Chemistry Transport Model CHIMERE accounting for meteorological conditions (temperature, humidity, and precipitation), gridded concentrations of NO_2_, PM_10,_ and PM_2.5_ (μg/m^3^) were produced with a spatial resolution approximately 4 km from 2000 to 2017 and 2 km since 2018 (17). These data provide a fine simulation of background levels but do not allow an accurate representation of pollution levels in the vicinity of specific local emission sources (dense road traffic areas and industrial areas). The model proposed by Real et al. in 2022 was yearly evaluated by a cross-validation process against background monitoring stations (rural, suburban, and urban) (17). They found high correlations and low root mean standard error (RMSE) for PM_10_ and PM_2.5_ in both rural and urban areas (Pearson’s correlation between 0.77 and 0.86; RMSE between 25 and 50% of the annual mean concentration). Pearson’s correlation was also high for NO_2_ (between 0.55 and 0.7 for rural areas and approximately 0.8 for urban areas), with background values in urban areas well represented (RMSE less than 25% of the annual mean concentration), while an overestimation of background concentrations was detected in rural areas (between 60 and 80% of the annual mean concentration).

The modeled annual average concentrations weighted by the population are given by INERIS at the municipality level as csv files for the different years. We considered these data for the 2009–2020 period as exposure indicators for each participant’s residential address at the time of dialysis initiation. These weighted data have been produced following European legislation on air quality monitoring for the mapping of geographical areas exceeding a limit value and the estimation of the number of inhabitants exposed to the exceedance. The spatial aggregation of air indicators by municipality is based on the gridded population data in proportion to the municipality’s area. The *i*^est^ municipality’s concentration weighted by the population ( $C_{\mathrm{iweighted}}$ ) is obtained by the following formula:

$$C_{\mathrm{iweighted}}=\frac{\sum_{\mathrm{grid}=1}^{N}C_{\mathrm{grid}}\times P_{\mathrm{grid}}}{\sum_{\mathrm{grid}=1}^{N}P_{\mathrm{grid}}}$$

with N the number of grid cells intersecting the municipality, $C_{\mathrm{grid}}$ the concentration in each grid cell of the municipality, and $P_{\mathrm{grid}}$ the population in the fraction of the grid cell corresponding to the municipality concerned. The population distribution data used by the French Central Air Quality Monitoring Laboratory to develop the population-weighted concentrations are those of 2015 and consider the spatial distribution of the inhabited areas within each municipality in France (18).

#### 2.2.2 Air pollution scores

In this study, we focused on air pollutants for which (i) reliable data were available nationwide during the study period (thus excluding SO_2_) and (ii) chronic exposure could be approximated by annual averages, as opposed to pollutants only present during peak periods (thus excluding ozone). In the end, the air pollution scores were constructed based on data concerning PM_2.5_, PM_10_, and NO_2_ (13, 15). Given the expected high correlation between the three air pollutants, we built a composite air pollution score by using the first component of a standardized principal component analysis (PCA) (15). The higher the score in a given municipality, the higher the overall level of air pollution. Annual air pollution PCA scores were generated based on annualized PCA. As the mathematical method used to build a composite index could affect air pollution scores’ results and interpretation, two other composite indices have been proposed for sensitivity analyses. The air pollution rank score is a cumulative ranking score, calculated according to a previously described formula (13, 14). Briefly, the score of the *i*^est^ municipality is obtained by the sum of the municipality’s ranks ( $n_{i}$ ) for each pollutant *p*.

$$\mathrm{AirPollutionScore}{\left(\mathrm{rank}\right)}_{i}=\overset{N}{\underset{p=1}{\sum }}n_{i,p}$$

The air pollution multiplicative impregnation ratio (MIR) score measures the synergistic effect of exceeding the World Health Organization’s Global Air Quality Guidelines (AQGs) for the three pollutants (19). It is obtained by multiplying the ratios of each pollutant’s annual concentration to their respective threshold for adverse health impact (5 μg/m^3^ for PM_2.5_, 15 μg/m^3^ for PM_10_, and 10 μg/m^3^ for NO_2_, respectively):


$$\mathrm{AirPollutionScore}{\left(MIR\right)}_{i}=\frac{C_{\mathrm{iweightedNO}2}}{AQG_{\mathrm{NO}2}}\times \frac{C_{\mathrm{iweightedPM}10}}{AQG_{\mathrm{PM}10}}\times \frac{C_{\mathrm{iweightedPM}2.5}}{AQG_{\mathrm{PM}2.5}}$$

#### 2.2.3 Temporal lag of exposure

As there was a strong decrease in annual average concentrations from 2009 to 2020 (48% for NO_2_, 41% for PM_10_, and 52% for PM_2.5_), we first considered the value for the year before starting dialysis, and second combined inter-annual average values for the 2 and 3 years prior to dialysis initiation (e.g., 2014, 2013–2014 average, and 2012–2014 average exposure value for a patient initiating dialysis in 2015).

### 2.3 Other environmental factors

Three ecological confounding factors were considered, and generated at the municipality scale. The socioeconomic deprivation index (FDep) was built from four variables in the INSEE database for the year 2015: the median household income, the percentage of high school graduates in the population aged 15 and over, the percentage of blue-collar workers in the active population, and the unemployment rate (20). The higher the FDep index, the greater the level of deprivation. The Euclidian distance (km) from the municipality centroid to the nearest hemodialysis center was used as an ecological proxy of healthcare access. The population density (inhabitant/km^2^) was also retrieved for each municipality. Given the association described in the literature between ambient temperature and the risk of cardiovascular mortality (21), all models were also adjusted for the annual average temperature of the year preceding the initiation of dialysis at the patient’s residence address. These data were derived from daily weather station observations from Météo France, the French national meteorological service (22, 23).

### 2.4 Outcomes

Causes of death were classified in prespecified categories according to the codes of the European Renal Association and the ICD-9 and ICD-10 coding systems (24). This classification has been previously described and standardized within the registry and is detailed in Supplementary Table S2 (25). Only the main causes of death were included in this study. Observations were censored at the date of the first event between death, kidney transplantation, loss to follow-up, and administrative censoring (31 December 2022).

### 2.5 Statistical analyses

Quantitative variables are expressed as means (standard deviation) in the case of normal distribution or medians (IQR) otherwise. Normality of distributions was assessed using histograms and the Shapiro–Wilk test. Categorical variables were expressed as numbers (percentages).

Considering kidney transplantation as a competitive risk, the association between air pollution scores and both all-cause mortality and cause-specific mortality (cardiovascular and infection-related) was analyzed using cause-specific Cox proportional hazards regression models adjusted for baseline characteristics: age, sex, body mass index, smoking status, comorbidities, mobility, dialysis care model (including peritoneal dialysis, in-center hemodialysis, self-care/home hemodialysis, and training center), conditions of dialysis initiation (functional dialysis access and emergency start), hemoglobin level (g/dL) and albumin level (g/L), the distance to the nearest dialysis center (km), the municipality population density (inhabitants/km^2^), and the social deprivation index (FDep). Cox proportional hazards regression models were also stratified by year (2012–2020) and dialysis center. Adjusted cumulative incidence curves by quartile of the air pollution score were derived from the Cox model using the G-formula method proposed by Denz *et al* (26). The HR and its 95% CI for each air pollution score have been derived from Cox models as effect size for one IQR increment. The proportional hazards assumption for each potential predictor was assessed by examining the scaled Schoenfeld residual plots, and the log-linearity assumption for each quantitative potential predictor was assessed by using Martingale residual plots. Effect sizes have been estimated after handling missing covariates by multiple imputation (27) using a regression switching approach [chained equations with *m* = 50 imputations; ‘*m*’ having been determined according to the method based on the fraction of missing data proposed by White et al. (28)]. Imputation was performed under the missing at-random assumption using all baseline characteristics with a predictive mean matching for continuous variables and logistic regression (binary, ordinal, or polynomial) for categorical variables. We therefore pooled the HRs from each imputed dataset using Rubin’s rules (29).

We further investigated the influence of age, sex, dialysis care model, the history of diabetes, the presence of cardiovascular comorbidities, the history of chronic respiratory disease, and the smoking status on the association between air pollution PCA score and all-cause mortality by including the corresponding interaction terms in the baseline characteristics adjusted Cox models.

### 2.6 Ethics and approvals

Data collection has been approved by the French National Commission for Information Technology and Privacy (CNIL, N° 903188), and patients are informed about their inclusion in the REIN registry. Data manually recorded from each dialysis center are then centralized in a national database where a record number, exclusive to the REIN registry, is given to each patient. The ‘*Agence de la biomédecine*’, a public institution, is REIN coordination body.

## 3 Results

**Figure 1:**
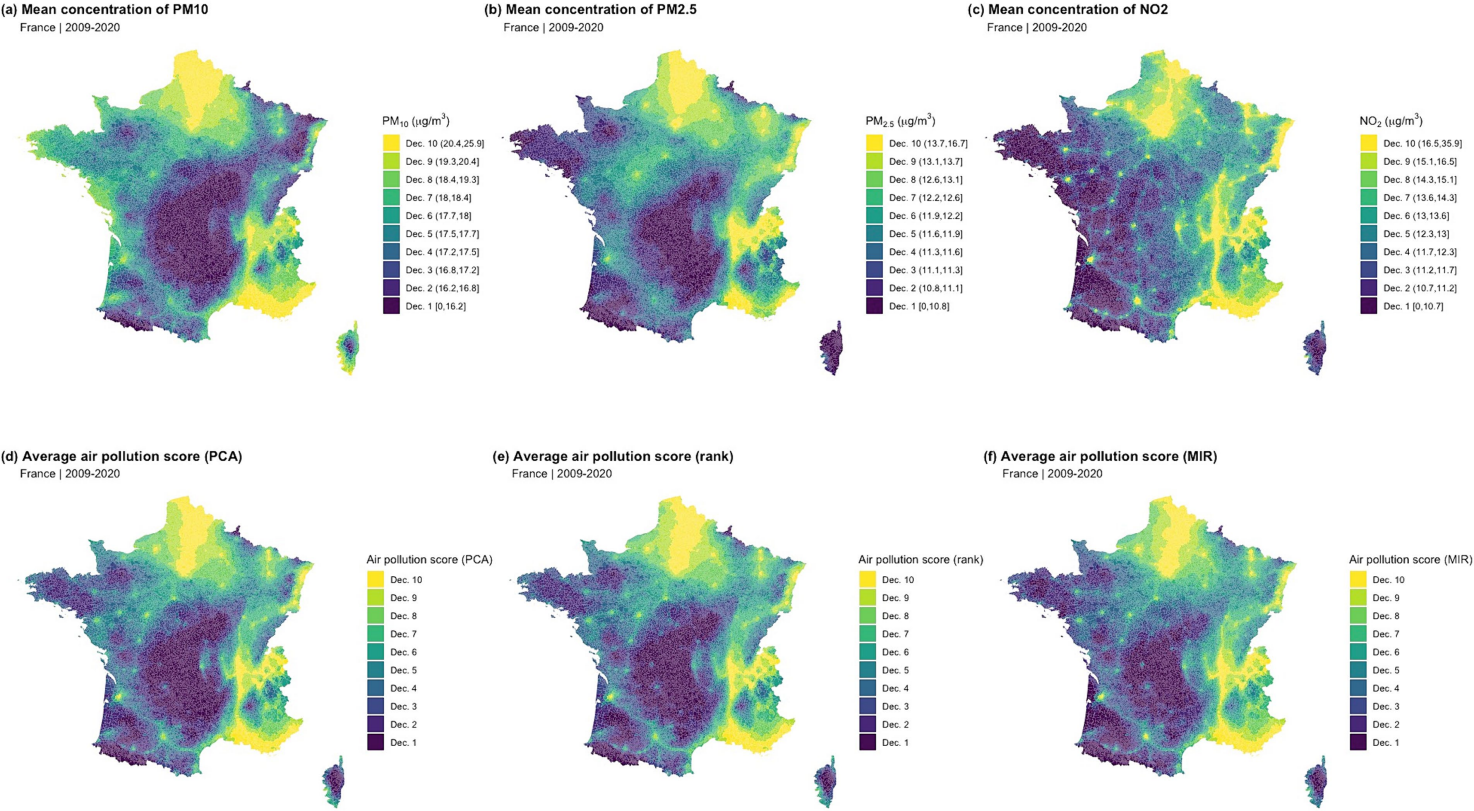
Spatial distribution of the individual air pollutants and composite air pollution scores (2009–2020 average). **(A)** Mean concentration of PM10; **(B)** Mean concentration of PM2.5; **(C)** Mean concentration of NO2; **(D)** Average air pollution score (PCA); **(E)** Average air pollution score (rank); **(F)** Average air pollution score (MIR)..

During the 2009–2020 period, the median weighted levels and ranges [min - max] for PM_2.5_, PM_10_, and NO_2_ in France were 11.9 [9.0–16.7] μg/m^3^, 17.7 [14.2–25.9] μg/m^3^, and 13.0 [8.6–36.0] μg/m^3^, respectively (Supplementary Figure S1, Supplementary Table S3). A strong correlation was observed among these air pollutants over the study period, estimated at 0.83, 0.76, and 0.66 for PM_2.5_-PM_10_, PM_2.5_-NO_2_, and PM_10_-NO_2_, respectively. The first principal component of the PCA, based on the values of the three air pollutants, explained an average of 83% of the variance (annual ranges from 69 to 84%, Supplementary Figure S2). The contributions of PM_2.5_, PM_10_, and NO_2_ to the air pollution PCA score were estimated at 36.9, 34.7, and 28.4%, respectively. Overall, a notable spatial heterogeneity in the distribution of the averaged air pollution scores over the study period was observed, with higher exposure zones located in proximity to major cities and roadways (Figure 1).

The cohort included a total of 90,373 incident dialysis patients between 2012 and 2020. This was a predominantly older adult population with a mean age of 69 years, primarily composed of male subjects (65%), and approximately half of the population suffering from diabetes (45%). The vast majority of the population (89%) received hemodialysis treatment, with over a quarter initiating treatment in an emergency setting (28%). The characteristics of the patients were generally comparable according to the different levels of the air pollution PCA score, except for the socio-environmental characteristics: the patients most exposed to air pollution were most likely living in municipalities with a higher population density, less socially deprived, and closer to the nearest dialysis center (Table 1).

**Table 1:** Baseline characteristics according to the quartiles of air pollution PCA score.

Characteristic	Missing (%)	Overall *N* = 90373^1^	Quartile 1 *N* = 22594^1^	Quartile 2 *N* = 22633^1^	Quartile 3 *N* = 22555^1^	Quartile 4 *N* = 22591^1^
Age (years)	0 (0.0%)	69 (15)	70 (14)	70 (14)	69 (15)	67 (16)
Male sex	0 (0.0%)	65% (58,469)	67% (15,116)	65% (14,656)	63% (14,238)	64% (14,459)
Body Mass Index (kg/m^2^)	14,567 (16%)					
<18.5		4.3% (3,259)	4.2% (866)	4.2% (841)	4.2% (788)	4.6% (764)
[18.5; 23]		23% (17,509)	23% (4,704)	23% (4,522)	22% (4,218)	25% (4,065)
[23; 25]		15% (11,659)	15% (3,155)	15% (3,046)	15% (2,897)	15% (2,561)
[25; 30]		32% (24,172)	33% (6,767)	32% (6,269)	32% (5,927)	31% (5,209)
> = 30		25% (19,207)	25% (5,090)	26% (5,165)	26% (4,981)	24% (3,971)
Smoking status	16,066 (18%)					
Never smoked		56% (41,463)	53% (9,992)	53% (9,915)	56% (10,255)	61% (11,301)
Currently or ex-smoking		44% (32,844)	47% (8,924)	47% (8,640)	44% (7,945)	39% (7,335)
History of diabetes	669 (0.7%)	45% (40,510)	43% (9,648)	45% (10,089)	46% (10,307)	47% (10,466)
Cardiovascular diseases	5,126 (5.7%)					
0–1		74% (62,798)	70% (14,860)	72% (15,296)	75% (15,981)	78% (16,661)
> = 2		26% (22,449)	30% (6,368)	28% (6,024)	25% (5,265)	22% (4,792)
History of chronic respiratory failure	2,521 (2.8%)	14% (12,120)	15% (3,237)	15% (3,205)	13% (2,913)	13% (2,765)
History of active cancer	2,098 (2.3%)	12% (10,345)	13% (2,852)	12% (2,696)	11% (2,347)	11% (2,450)
History of cirrhosis	2,145 (2.4%)	2.7% (2,415)	2.9% (647)	2.8% (613)	2.6% (578)	2.6% (577)
History of severe behavioral disorders	6,629 (7.3%)	3.0% (2,492)	3.1% (656)	3.2% (661)	2.8% (567)	2.9% (608)
Mobility	7,493 (8.3%)					
Totally dependent		4.8% (3,967)	3.7% (783)	4.4% (908)	5.4% (1,112)	5.7% (1,164)
Need assistance		12% (10,067)	10% (2,200)	11% (2,256)	12% (2,537)	15% (3,074)
Walk without help		83% (68,846)	86% (18,404)	85% (17,639)	82% (16,756)	79% (16,047)
Dialysis care model	0 (0.0%)					
Peritoneal dialysis		11% (9,683)	12% (2,677)	12% (2,667)	11% (2,434)	8.4% (1,905)
In-center hemodialysis		82% (73,898)	79% (17,796)	81% (18,319)	83% (18,785)	84% (18,998)
Self-care/home hemodialysis		3.7% (3,358)	2.3% (520)	3.1% (700)	3.7% (829)	5.8% (1,309)
Training center		3.8% (3,434)	7.1% (1,601)	4.2% (947)	2.2% (507)	1.7% (379)
Emergency start	4,259 (4.7%)	28% (24,525)	26% (5,631)	28% (6,021)	28% (6,027)	32% (6,846)
Hemoglobin level (g/dL)	11,104 (12%)					
<10		47% (37,527)	44% (8,827)	47% (9,197)	48% (9,504)	51% (9,999)
(10; 12)		40% (32,103)	42% (8,553)	41% (8,065)	40% (7,796)	39% (7,689)
> = 12		12% (9,639)	14% (2,781)	13% (2,489)	12% (2,355)	10% (2,014)
Albumin level (g/L)	27,602 (31%)					
<30		27% (16,700)	26% (4,119)	27% (4,174)	26% (4,054)	28% (4,353)
(30, 35)		29% (18,033)	28% (4,584)	29% (4,469)	28% (4,353)	29% (4,627)
> = 35		45% (28,038)	46% (7,390)	45% (6,982)	45% (6,939)	43% (6,727)
Distance to nearest dialysis center (km)	375 (0.4%)	10 (11)	19 (13)	12 (11)	8 (8)	3 (3)
Deprivation index (FDep)	271 (0.3%)					
Quintile 1		20% (18,057)	9.3% (2,076)	17% (3,836)	23% (5,096)	31% (7,049)
Quintile 2		20% (17,991)	25% (5,555)	22% (5,018)	19% (4,381)	13% (3,037)
Quintile 3		21% (19,176)	27% (6,012)	19% (4,326)	16% (3,602)	23% (5,236)
Quintile 4		19% (16,902)	25% (5,631)	21% (4,816)	17% (3,778)	12% (2,677)
Quintile 5		20% (17,976)	14% (3,152)	20% (4,566)	25% (5,667)	20% (4,591)
Population density (inhabitants/km^2^)	271 (0.3%)	2,639 (4,623)	327 (602)	860 (1,159)	1,659 (1,696)	7,686 (6,767)

^1^mean (standard deviation) for quantitative variables; %(no.) for categorical variables.

**Figure 2:**
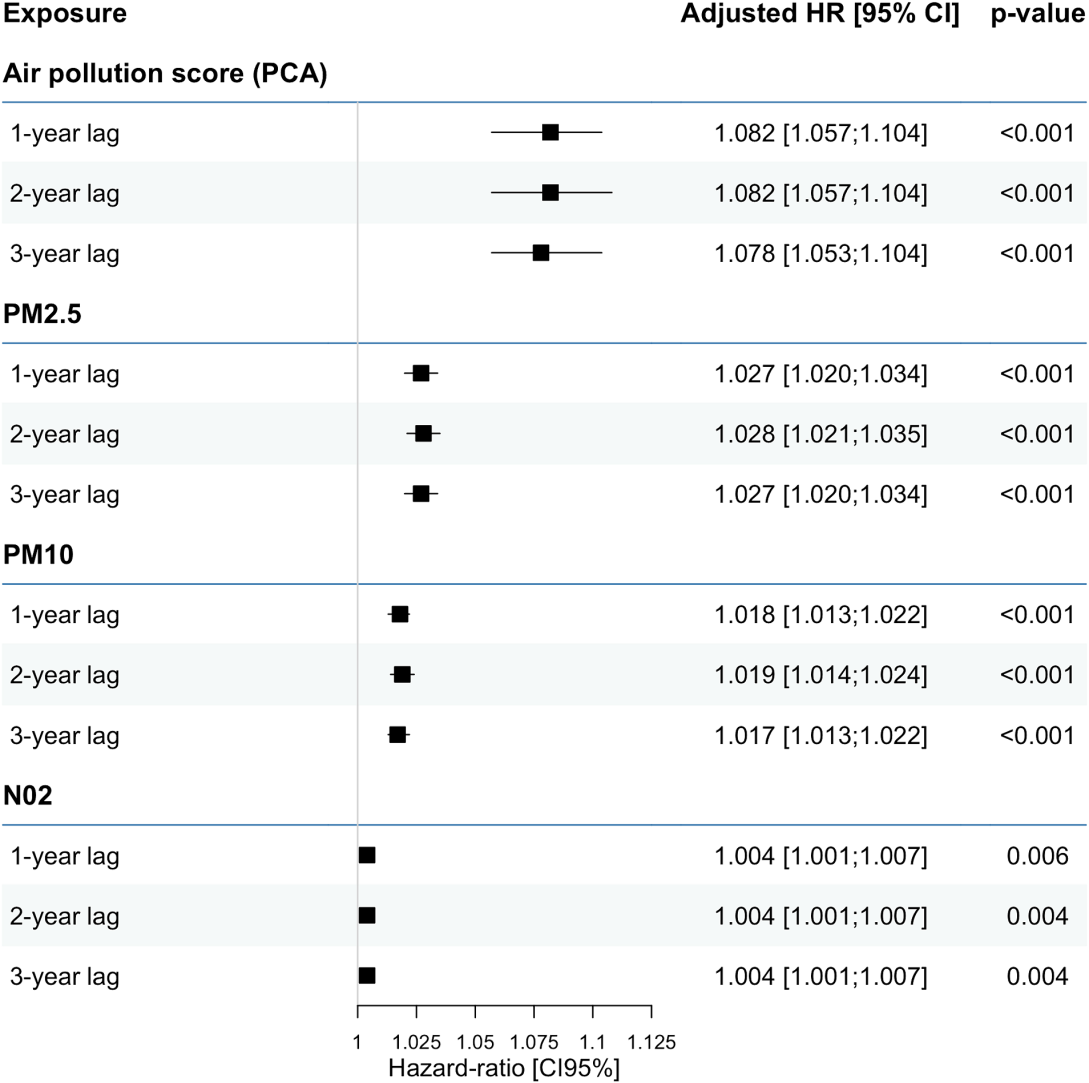
Association between air pollution PCA score and risk of all-cause mortality.

Over a median follow-up period of 27 months, a total of 44,242 deaths were observed, of which 8,859 were attributed to cardiovascular causes (20.0%) and 6,860 to infectious causes (15.5%). After adjusting for baseline characteristics, we observed a progressive increase in the cumulative incidence of all-cause mortality with quartiles of the air pollution PCA score (*p* < 0.001, Supplementary Figure S3). A significant linear association was thus found between air pollution exposure level and all-cause mortality, both in the analyses using the air pollution PCA score and considering each of the three main pollutants individually. An IQR increment in the air pollution PCA score was associated with an approximately 8.2% increase in the hazard of all-cause mortality (HR, 1.082 [95% confidence interval, 1.057–1.104]). This association appeared to be more pronounced with a rise in PM_2.5_ levels, where a 1 μg/m^3^ elevation was associated with an excess hazard of all-cause mortality estimated at HR, 1.027 [95% CI, 1.020–1.034]. These results remained consistent regardless of the exposure lag, ranging from 1 to 3 years (Figure 2).

Similarly, a significant association was observed between air pollution and both the risks of cardiovascular and infectious causes of mortality. Notably, the association appeared to be stronger for infectious cause mortality compared to cardiovascular mortality, as evidenced by HRs of 1.686 [95% CI, 1.470–1.932] and 1.212 [95% CI, 1.074–1.368], respectively, for an IQR increment of the air pollution PCA score (Supplementary Table S4). Consistent findings were observed in the analyses focusing on individual pollutants, wherein the risk of infectious mortality exhibited a higher magnitude than that of cardiovascular mortality. Specifically, HR demonstrated an elevated risk of both cardiovascular and infection-related mortality for PM_2.5_ and PM_10_ exposure, while statistically significant associations were found between NO_2_ levels and infectious mortality only.

**Figure 3:**
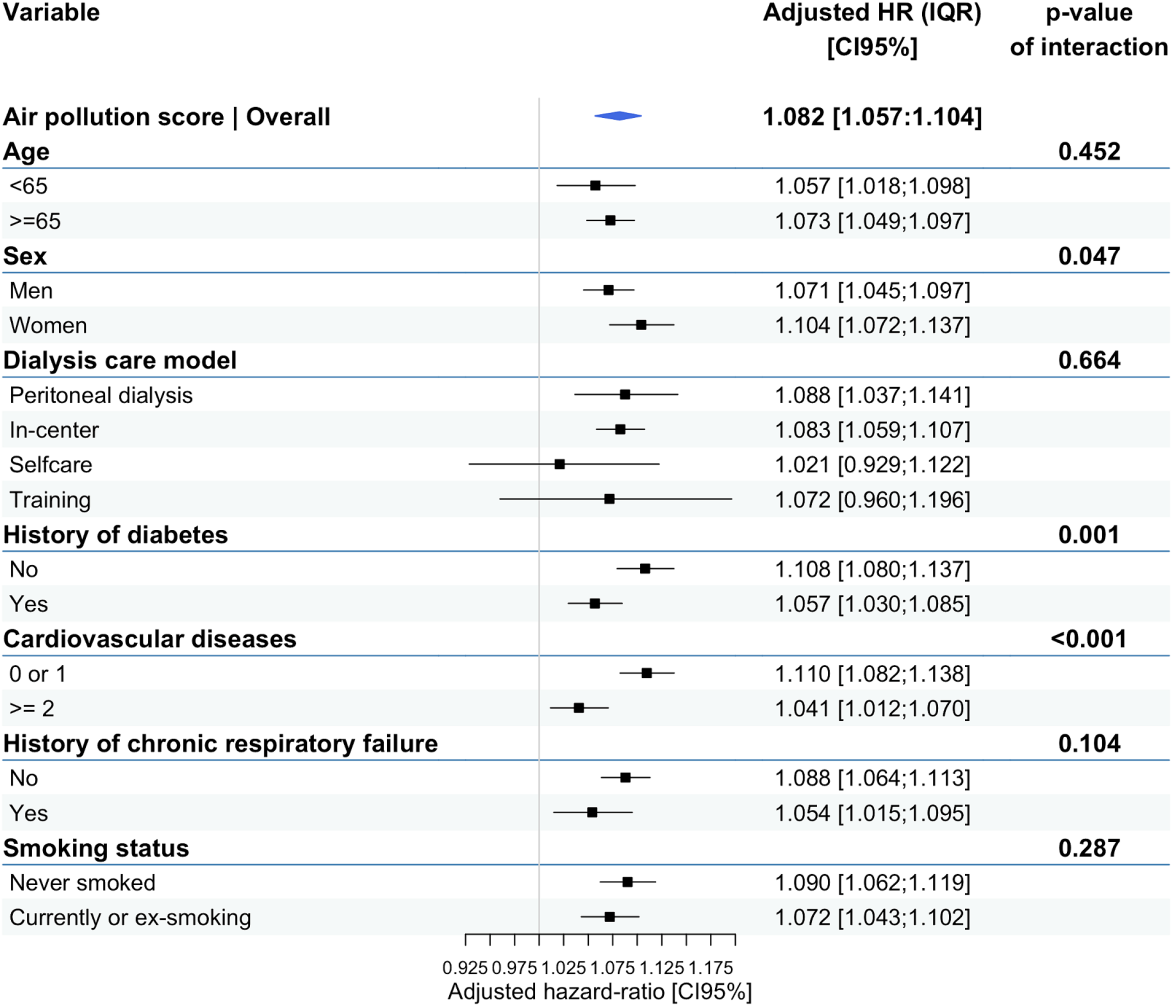
Effect-modifying analyses: association between air pollution PCA score and all-cause mortality according to different subgroups.

The analysis of interactions yielded consistent results across different subgroups of the population: all-cause and cause-specific mortality appeared significantly associated with the air pollution score (Figure 3). However, these associations appeared slightly stronger in women and non-diabetic patients with minimal or no cardiovascular comorbidities. Conversely, the observed associations were not influenced by the dialysis care model, smoking status, or history of chronic respiratory disease.

The results remained largely consistent in sensitivity analyses involving alternative composite air pollution scores (Supplementary Figures S4–S6). The air pollution rank score highlighted a linear association between the risk of all-cause mortality and infection-related deaths.

## 4 Discussion

This study is the first to our knowledge to demonstrate an increased risk of all-cause mortality for the two main causes of death in dialysis patients (cardiovascular and infection-related), in association with multiple air pollutant exposures. We thus observe an 8.2% increased hazard of all-cause mortality for each IQR increment of the combined air pollution PCA score. This association seems even stronger for deaths from infectious causes, with an estimated 68.6% excess hazard for each IQR increase in air pollution exposure. These results are globally consistent, regardless of the exposure lag and the alternative composite air pollution scores. Finally, certain patient profiles appear to be more vulnerable to the effects of environmental exposure, notably women and less comorbid patients.

Based on the recent literature, we were able to identify overall 10 studies investigating the association between air pollution exposure and dialysis mortality over the last decade, with variable designs, population sizes, and characteristics (10, 30–38) (Supplementary Table S5). None of these studies were conducted in Europe, the vast majority having been carried out in the USA and Taiwan. Of these studies, only six investigated the link between long-term exposure to air pollution and the risk of death in dialysis, with none considering multi-exposure (analyses mainly based on PM_2.5_ exposure) (10, 30–32, 36, 38). Despite the potential for publication bias, all these studies seem to agree on a significant association between air pollution and dialysis mortality. In the study carried out by Xi Y. et al. in 2022 on a population with characteristics similar to our own (314,079 US patients, mean age 64 years), the authors highlighted a comparable effect size, with an excess hazard of death from cardiovascular causes of approximately 2% per 1 μg/m^3^ increment of PM_2.5_ estimated at the dialysis center’s ZIP code (10). Our nationwide study thus stands out for its estimation of long-term multi-exposure to air pollution combining the three main pollutants at the patients’ residential place. These results are reinforced by the different approaches used to build the composite environmental scores, providing overall consistent results.

Another unique aspect of our research revolves around the analysis of both specific cardiovascular and infectious-related mortality, which accounts for more than one-third of deaths within our study population. The link between air pollution and cardiovascular events, all-cause mortality, and cardiovascular mortality now appears to be well-established in the literature, including in the general population (39). The dialysis patient population, despite an already increased risk of cardiovascular events due to kidney failure, also appears to be concerned by the potential influence of environmental exposure on the risk of cardiovascular events (10, 33). In their study, Xi Y. et al. also concluded that each 1 μg/m^3^ greater annual average PM_2.5_ was associated with a 2% greater rate of cardiovascular events in US adult kidney failure patients initiating hemodialysis (10). Numerous pathophysiological explanations have already been suggested, including pro-inflammatory state, endothelial dysfunction, increased blood viscosity, cardiac autonomic system dysregulation, and other potential mechanisms inducing direct ischemia or arrhythmogenesis (39–41). One of the original features of our study lies in the identification of an additional risk of death from infectious causes, which, to our knowledge, has not been previously reported. In our study, we observed a marked excess risk of death from infectious causes, with a size effect appearing much greater than that observed for cardiovascular mortality. This original result could be explained by the potential immune dysfunction related to air pollution, in an already vulnerable population subject to relative immunodepression due to kidney failure (4, 42). Indeed, patients with kidney failure display impaired naïve and acquired immune systems related to multifactorial causes such as uremic toxins, malnutrition, and chronic inflammation, contributing to a high prevalence of infections and making sepsis the second leading cause of mortality in patients undergoing dialysis (43). Recent literature appears to highlight a strong association between air pollution exposure and infectious-related mortality: a recent study based on a cohort of over 53 million US Medicare beneficiaries observed a 9% increase in sepsis-related mortality linked to a 10 μg/m^3^ rise in the 12-month moving average of PM_2.5_ (44). As also suggested by the recent results of the ELAPSE multicenter project, which involved over 300,000 people among six European countries, long-term exposure to air pollution was associated with an increased risk of pneumonia-related mortality (45). This may explain our results since bloodstream and respiratory infections represent two major causes of infection in the dialysis patient population (46, 47).

Our findings also suggest larger magnitudes of association between sub-chronic air pollutant exposure and health outcomes among women, and patients who were not diabetic or had fewer cardiovascular diseases at dialysis initiation. A trend, albeit not statistically significant, was also noted toward a more pronounced effect in older adult subjects. Sex disparities in the effect of air pollution on cardiovascular disease risk have been previously documented in the literature, yet results remain inconclusive (48). Some studies suggest an increased vulnerability in women, while others suggest the opposite. No definitive conclusions can be drawn in the current case, especially given the relatively moderate difference in effect size observed in our study. Interestingly, the effect size appears to be more pronounced in patients with fewer comorbidities at the initiation of dialysis, a finding consistent with previous observations (10). One potential explanation could be the relatively limited influence of environmental exposure compared to other risk factors more strongly associated with mortality in the most at-risk subgroups. Finally, the lack of interaction with the dialysis care model is in line with existing literature, as the association between air pollution and mortality holds in studies involving both peritoneal dialysis and hemodialysis patients (10, 32, 38).

This study does have certain limitations. First, due to their observational design, these results are subject to potential residual confounding. These results specifically concern the French population of dialysis patients, which might limit generalizability to other regions. However, these data are important as this is, to our knowledge, the first nationwide study of this kind in Europe. Furthermore, the literature indicates similar associations in magnitude in other populations exposed to comparable pollution levels, thus supporting external validity (10). Exposure data are based on annual average concentrations, providing an approximation of the municipality-level background pollution with a potential risk of exposure misclassification. Nonetheless, these air pollutants are generally regarded and regulated on a regional basis, and exposure modeling currently stands as the best available option at the national level (15, 17). The literature review carried out as part of this study (Supplementary Table S5) also highlighted that this is a relevant measurement scale in this context, assuming that patients with advanced kidney disease rarely change their place of residence. Furthermore, these data appear suitable for estimating sub-chronic exposure, as evidenced by consistent results across various temporal lags. Dialysis patients are more likely to spend time indoors, increasing their exposure to indoor pollutants. However, many studies have confirmed that indoor air quality is highly influenced by outdoor air quality (49). Therefore, it is likely that we are non-differentially underestimating the actual exposure levels and their health effects for our entire study population. Furthermore, the present data do not allow for more details on the type of cardiovascular or infectious events leading to patient deaths, warranting further investigations. Moreover, potential ethnic disparities in the air pollution effect could not be explored, as the collection of such individual data is prohibited in France. This study did not explore the assessment of risks associated with short-term combined environmental exposure and should also be the subject of future research endeavors, as well as the development of tools for assessing individual and indoor air pollution exposure.

## 5 Conclusion

In summary, our nationwide registry-based study provides compelling evidence of a significant association between multiple air pollutants and both cardiovascular and infectious cause mortality in incident dialysis patients. Moreover, the differential impact on infectious cause mortality underscores the need for targeted interventions to mitigate the adverse health effects of air pollution in this highly vulnerable population.

## Data availability statement

The raw data supporting the conclusions of this article will be made available by the authors, without undue reservation.

## Ethics statement

The studies involving humans were approved by French National Commission for Information Technology and Privacy (CNIL, N° 903188). The studies were conducted in accordance with the local legislation and institutional requirements. Written informed consent for participation was not required from the participants or the participants’ legal guardians/next of kin in accordance with the national legislation and institutional requirements.

## Author contributions

AH: Conceptualization, Data curation, Formal analysis, Funding acquisition, Methodology, Project administration, Resources, Software, Supervision, Validation, Visualization, Writing – original draft, Writing – review & editing. MG: Conceptualization, Formal analysis, Methodology, Software, Supervision, Validation, Visualization, Writing – original draft, Writing – review & editing. FG: Investigation, Supervision, Writing – review & editing. BS: Supervision, Validation, Writing – review & editing. KL: Methodology, Supervision, Validation, Writing – review & editing. AC: Data curation, Formal analysis, Methodology, Software, Validation, Writing – original draft, Writing – review & editing. LD: Methodology, Supervision, Validation, Writing – review & editing. VG: Methodology, Supervision, Validation, Writing – review & editing. FB: Data curation, Methodology, Validation, Writing – review & editing. ML: Data curation, Methodology, Validation, Writing – review & editing. CC: Data curation, Methodology, Software, Supervision, Validation, Writing – review & editing. PA: Methodology, Supervision, Validation, Writing – review & editing. FO: Data curation, Formal analysis, Methodology, Supervision, Validation, Visualization, Writing – original draft, Writing – review & editing.
