# Supplementary material for: Multiple air pollutant exposure is associated with higher risk of all-cause mortality in dialysis patients: a French registry-based nationwide study
Source: Front Public Health. 2024 Jul 30;12:1390999. doi: 10.3389/fpubh.2024.1390999 (PMC11319261; doi:10.3389/fpubh.2024.1390999)
Supplement: Supplementary file 1 [file Data_Sheet_1.PDF]

Supplementary Material

Title

Multiple air pollutant exposure is associated with higher risk of all-cause mortality in dialysis patients: a French registry-based nationwide study

Authors

Aghiles Hamroun, Michaël Génin, François Glowacki, Bénédicte Sautenet, Karen Leffondré, Antoine De Courrèges, Luc Dauchet, Victoria Gauthier, Florian Bayer, Mathilde Lassalle, Cécile Couchoud, Philippe Amouyel, Florent Occelli

Table of content

Table S1. STROBE Checklist.
Table S2. ERA-EDTA cause of death codes for cardiovascular and infection-related mortality.
Table S3. Average air pollutants' concentration levels and air pollution scores in France (2009-2020).
Table S4. Association between air pollution PCA score (and individual air pollutant concentration levels) and risk of cause-specific mortality.
Table S5. Air pollution and mortality risk in dialysis patients – review of the current evidence.

Figure S1. Distribution of average concentration levels of each air pollutant and pollution scores (2009-2020).
Figure S2. Construction of the air pollution PCA score: (a) bivariate Pearson's correlation between air pollutants ($PM_{2.5}$, PM_{10}, NO_2), (b) correlation circle from the principal component analysis.
Figure S3. Adjusted cumulative incidence of all-cause mortality according to the air pollution PCA score (quartiles).
Figure S4. Sensitivity analysis: association between multiple air pollutant exposure and risk of all-cause mortality, according to the alternative composite air pollution scores.
Figure S5. Sensitivity analysis: association between multiple air pollutant exposure and risk of cardiovascular mortality, according to the alternative composite air pollution scores.
Figure S6. Sensitivity analysis: association between multiple air pollutant exposure and risk of infection-related mortality, according to the alternative composite air pollution scores.

Table S1. STROBE Checklist

	Item No	Recommendation	Page
**Title and abstract**	**1**	(*a*) Indicate the study's design with a commonly used term in the title or the abstract<br>(*b*) Provide in the abstract an informative and balanced summary of what was done and what was found	**1**<br>**2**
**Introduction**			
Background/rationale	**2**	Explain the scientific background and rationale for the investigation being reported	**3-4**
Objectives	**3**	State specific objectives, including any prespecified hypotheses	**4**
**Methods**			
Study Design	**4**	Present key elements of study design early in the paper	**5**
Setting	**5**	Describe the setting, locations, and relevant dates, including periods of recruitment, exposure, follow-up, and data collection	**5-8**
Participants	**6**	(*a*) Give the eligibility criteria, and the sources and methods of selection of participants. Describe methods of follow-up<br>(*b*) For matched studies, give matching criteria and number of exposed and unexposed	**5**
Variables	**7**	Clearly define all outcomes, exposures, predictors, potential confounders, and effect modifiers. Give diagnostic criteria, if applicable	**5-8**
Data sources/ measurement	**8***	For each variable of interest, give sources of data and details of methods of assessment (measurement). Describe comparability of assessment methods if there is more than one group	**5-8**
Bias	**9**	Describe any efforts to address potential sources of bias	**5-8**
Study size	**10**	Explain how the study size was arrived at	**-**
Quantitative variables	**11**	Explain how quantitative variables were handled in the analyses. If applicable, describe which groupings were chosen and why	**9-10**
Statistical methods	**12**	(*a*) Describe all statistical methods, including those used to control for confounding<br>(*b*) Describe any methods used to examine subgroups and interactions<br>(*c*) Explain how missing data were addressed<br>(*d*) If applicable, explain how loss to follow-up was addressed<br>(*e*) Describe any sensitivity analyses	**9-10**
**Results**			
Participants	**13***	*(a)* Report numbers of individuals at each stage of study—eg numbers potentially eligible, examined for eligibility, confirmed eligible, included in the study, completing follow-up, and analyzed<br>*(b)* Give reasons for non-participation at each stage<br>*(c)* Consider use of a flow diagram	**11**
Descriptive data	**14***	*(a)* Give characteristics of study participants (eg demographic, clinical, social) and information on exposures and potential confounders<br>*(b)* Indicate number of participants with missing data for each variable of interest<br>*(c)* Summarize follow-up time (eg, average and total amount)	**11**<br>**Tab1**
Outcome data	**15***	Report numbers of outcome events or summary measures over time	**11, FigS3**
Main results	**16**	(*a*) Give unadjusted estimates and, if applicable, confounder-adjusted estimates and their precision (eg, 95% confidence interval). Make clear which confounders were adjusted for and why they were included<br>(*b*) Report category boundaries when continuous variables were categorized<br>(*c*) If relevant, consider translating estimates of relative risk into absolute risk for a meaningful time period	**12-13**<br>**Fig2**<br>**TabS4**
Other analyses	**17**	Report other analyses done—eg analyses of subgroups and interactions, and sensitivity analyses	**12-13**<br>**Fig3**<br>**Fig S4-S6**
**Discussion**			
Key results	**18**	Summarize key results with reference to study objectives	**14**

Limitations	**19**	Discuss limitations of the study, taking into account sources of potential bias or imprecision. Discuss both direction and magnitude of any potential bias	**16-17**
Interpretation	**20**	Give a cautious overall interpretation of results considering objectives, limitations, multiplicity of analyses, results from similar studies, and other relevant evidence	**15-17**<br>**TabS5**
Generalizability	**21**	Discuss the generalizability (external validity) of the study results	**16-17**
**Other information**			
Funding	**22**	Give the source of funding and the role of the funders for the present study and, if applicable, for the original study on which the present article is based	**23**

*Give information separately for exposed and unexposed groups.

Table S2. ERA-EDTA cause of death codes for cardiovascular and infection-related mortality.

Category	Cause of death (related code)
**Cardiovascular**	Myocardial ischemia and infarction (11)<br>Hemorrhagic pericarditis (13)<br>Other causes of cardiac failure (14)<br>Cardiac arrest, cause unknown (15)<br>Hypertensive cardiac failure (16)<br>Fluid overload (18)<br>Pulmonary embolus (21)<br>Cerebrovascular accident (22)<br>Mesenteric infarction (29)
**Infection**	Bacterial pulmonary infection (31)<br>Viral pulmonary infection (32)<br>Fungal or protozoal pulmonary infection (33)<br>Infections elsewhere except viral hepatitis (34)<br>Septicemia (35)<br>Tuberculosis(lung) (36)<br>Tuberculosis (elsewhere) (37)<br>Generalized viral infection (38)<br>Liver disease due to Hepatitis B virus (41)<br>Liver disease due to other viral hepatitis (42)

Table S3. Average air pollutants' concentration levels and environmental scores in France (2009-2020)

Variable	Missing (%)	Overall[1]
**$PM_{2.5}$**	0 (0%)	
Mean (SD)		12.08 (1.13)
Median [25%; 75%]		11.92 [11.22; 12.79]
Range		9.03, 16.72
**PM_{10}**	0 (0%)	
Mean (SD)		18.00 (1.60)
Median [25%; 75%]		17.70 [16.99; 18.84]
Range		14.18, 25.93
**NO_2**	0 (0%)	
Mean (SD)		13.39 (2.65)
Median [25%; 75%]		12.97 [11.41; 14.70]
Range		8.59, 35.93
**PCA score**	0 (0%)	
Mean (SD)		0.00 (1.42)
Median [25%; 75%]		-0.34 [-0.98; 0.78]
Range		-2.90, 7.59
**Rank score**	0 (0%)	
Mean (SD)		52,932 (24,640)
Median [25%; 75%]		48,675 [33,397; 73,253]
Range		3,691, 105,252
**Rim score**	0 (0%)	
Mean (SD)		4.47 (1.66)
Median [25%; 75%]		4.00 [3.27; 5.21]
Range		2.01, 17.93

[1]mean (standard deviation) or median [IQR] for quantitative variables; no·/total no· (%) for categorical variables

Table S4. Association between air pollution PCA score (and individual air pollutant concentration levels) and risk of cause-specific mortality.

Exposure	Cause-specific death	Exposure lag	HR [95% CI]*	*p-value*
**Score PCA**	Cardiovascular	1 year	1.2118 [1.0744;1.3676]	0.002
	Infection	1 year	1.6856 [1.4703;1.9325]	<0.001
	Cardiovascular	2 years	1.193 [1.0543;1.3491]	0.005
	Infection	2 years	1.6792 [1.459;1.9325]	<0.001
	Cardiovascular	3 years	1.164 [1.0263;1.3193]	0.018
	Infection	3 years	1.7065 [1.4802;1.9672]	<0.001
**$PM_{2.5}$**	Cardiovascular	1 year	1.0259 [1.0116;1.0404]	<0.001
	Infection	1 year	1.0669 [1.0496;1.0844]	<0.001
	Cardiovascular	2 years	1.0263 [1.0113;1.0415]	0.001
	Infection	2 years	1.0669 [1.0488;1.0853]	<0.001
	Cardiovascular	3 years	1.023 [1.0079;1.0383]	0.003
	Infection	3 years	1.0698 [1.0515;1.0884]	<0.001
**PM_{10}**	Cardiovascular	1 year	1.0129 [1.0025;1.0235]	0.015
	Infection	1 year	1.0448 [1.0324;1.0573]	<0.001
	Cardiovascular	2 years	1.0125 [1.0016;1.0234]	0.024
	Infection	2 years	1.044 [1.0311;1.057]	<0.001
	Cardiovascular	3 years	1.0094 [0.9985;1.0203]	0.090
	Infection	3 years	1.0442 [1.0313;1.0573]	<0.001
**NO_2**	Cardiovascular	1 year	1.0052 [0.9991;1.0113]	0.098
	Infection	1 year	1.0136 [1.0068;1.0205]	<0.001
	Cardiovascular	2 years	1.0053 [0.9991;1.0115]	0.092
	Infection	2 years	1.0144 [1.0076;1.0214]	<0.001
	Cardiovascular	3 years	1.0046 [0.9985;1.0108]	0.140
	Infection	3 years	1.0147 [1.0078;1.0216]	<0.001

HR: hazard ratio; 95%CI: 95% confidence interval

**HR expressed per IQR increment for PCA score and per 1µg/m³ increment for $PM_{2.5}$, PM_{10}, and NO_2.*

Table S5. Air pollution and mortality risk in dialysis patients – review of the current evidence.

Author (year)	Country	Study design	Size, period	Population characteristics	Exposure, mean $PM_{2.5}$ level	Main result
Huang W-H *et al* (2013)[33]	Taiwan	Prospective cohort study	N = 256 2009-2011	Elderly HD patients Age (mean) 73y Sex ratio (M/F) 50≈50	Comparison of two living aeras (Taipei) mean $PM_{2.5}$ 25-30 μg/m³	Higher risk of 2-year all-cause mortality
Lin J-H *et al* (2015)[35]	Taiwan	Retrospective cohort study	N = 160 2009-2011	Adult PD patients Age (mean) 50y Sex ratio (M/F) 25≈75	Long-term $PM_{2.5}$, PM_{10}, NO_2, and SO_2 exposure at the district-level mean $PM_{2.5}$ 29.6 μg/m³	Higher risk of 2-year mortality (only NO_2)
Jung J *et al* (2020)[30]	Korea	Prospective cohort study	N = 5,041 2008-2015	Dialysis patients Age (mean) 61y Sex ratio (M/F) 60≈40	Long-term PM_{10}, NO_2, and SO_2 exposure at the province-level mean PM_{10} 55 μg/m³	Higher risk of all-cause mortality
Xi Y *et al* (2020)[34]	USA	Retrospective time-series analysis	N = 48,454 (cases) 2008-2012	Adult HD patients Age (mean) 65y Sex ratio (M/F) 50≈50	Short-term wildfire-$PM_{2.5}$ exposure at the county level (dialysis clinics) mean wildfire-$PM_{2.5}$ 1.01 μg/m³	Higher risk of all-cause mortality
Feng Y *et al* (2021)[29]	USA	Retrospective cohort study	N = 384,276 2010-2016	Older dialysis patients Age (mean) 74y Sex ratio (M/F) 85≈15	Long-term $PM_{2.5}$ exposure at the ZIP-code level mean $PM_{2.5}$ 9.2 μg/m³	Higher risk of all-cause mortality
Xi Y *et al* (2022)[10]	USA	Retrospective cohort study	N = 314,079 2011-2016	Adult HD patients Age (mean) 64y Sex ratio (M/F) 50≈50	Long-term $PM_{2.5}$ exposure at the ZIP-code level (dialysis clinics) mean $PM_{2.5}$ 8.8 μg/m³	Higher risks of cardiovascular events and mortality
Xi Y *et al* (2022)[32]	USA	Retrospective cohort study	N = 314,079 2011-2016	Adult HD patients Age (mean) 65y Sex ratio (M/F) 50≈50	Short-term $PM_{2.5}$ exposure at the ZIP-code level (dialysis clinics) mean $PM_{2.5}$ 8.6 μg/m³	Higher risks of cardiovascular events and mortality
Lou X *et al* (2022)[36]	China	Time-stratified case-crossover study	N = 18,114 (cases) 2013-2020	Dialysis patients Age (median) 68y Sex ratio (M/F) 60≈40	Short-term $PM_{2.5}$, PM_{10} exposure (dialysis clinics) mean $PM_{2.5}$ 44.4 μg/m³	Higher risk of all-cause mortality
Chen SF *et al* (2023)[31]	Taiwan	Retrospective population-based cohort study	N = 34,088 2000-2013	Dialysis patients Age (mean) 59y Sex ratio (M/F) 50≈50	Long-term $PM_{2.5}$ exposure at the ZIP-code level median $PM_{2.5}$ 31.2 μg/m³	Higher risk of all-cause mortality
Hu S *et al* (2023)[37]	China	Retrospective cohort study	N = 886 2013-2018	Adult PD patients Age (mean) 57y Sex ratio (M/F) 50≈50	Long-term $PM_{2.5}$, PM_{10}, NO_2, and SO_2 exposure at the district level median $PM_{2.5}$ 58 μg/m³	Higher risk of all-cause mortality

HD: hemodialysis; PD: peritoneal dialysis; M/F: male/female

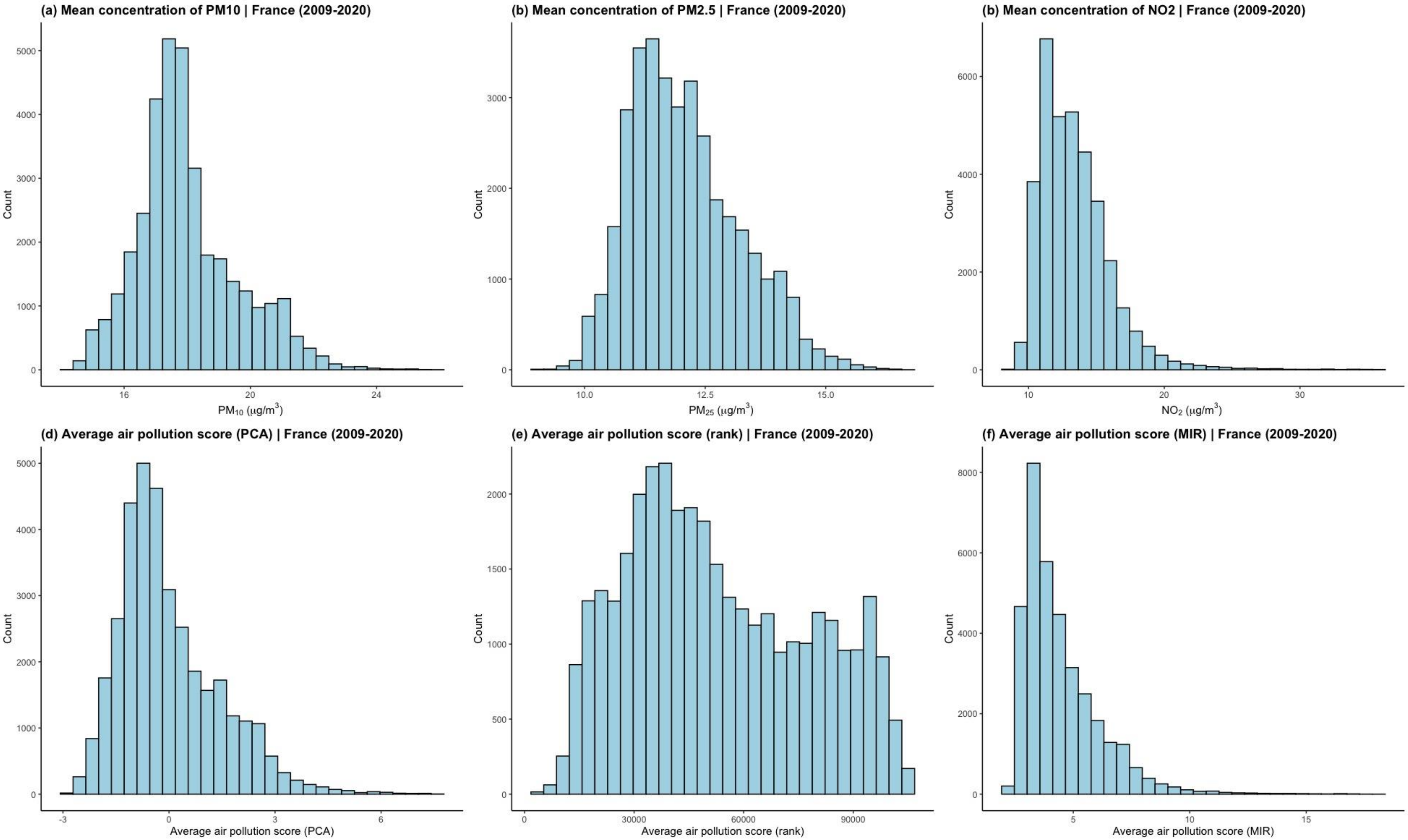

Figure S1. Distribution of average concentration levels of each air pollutant and pollution scores (2009-2020).

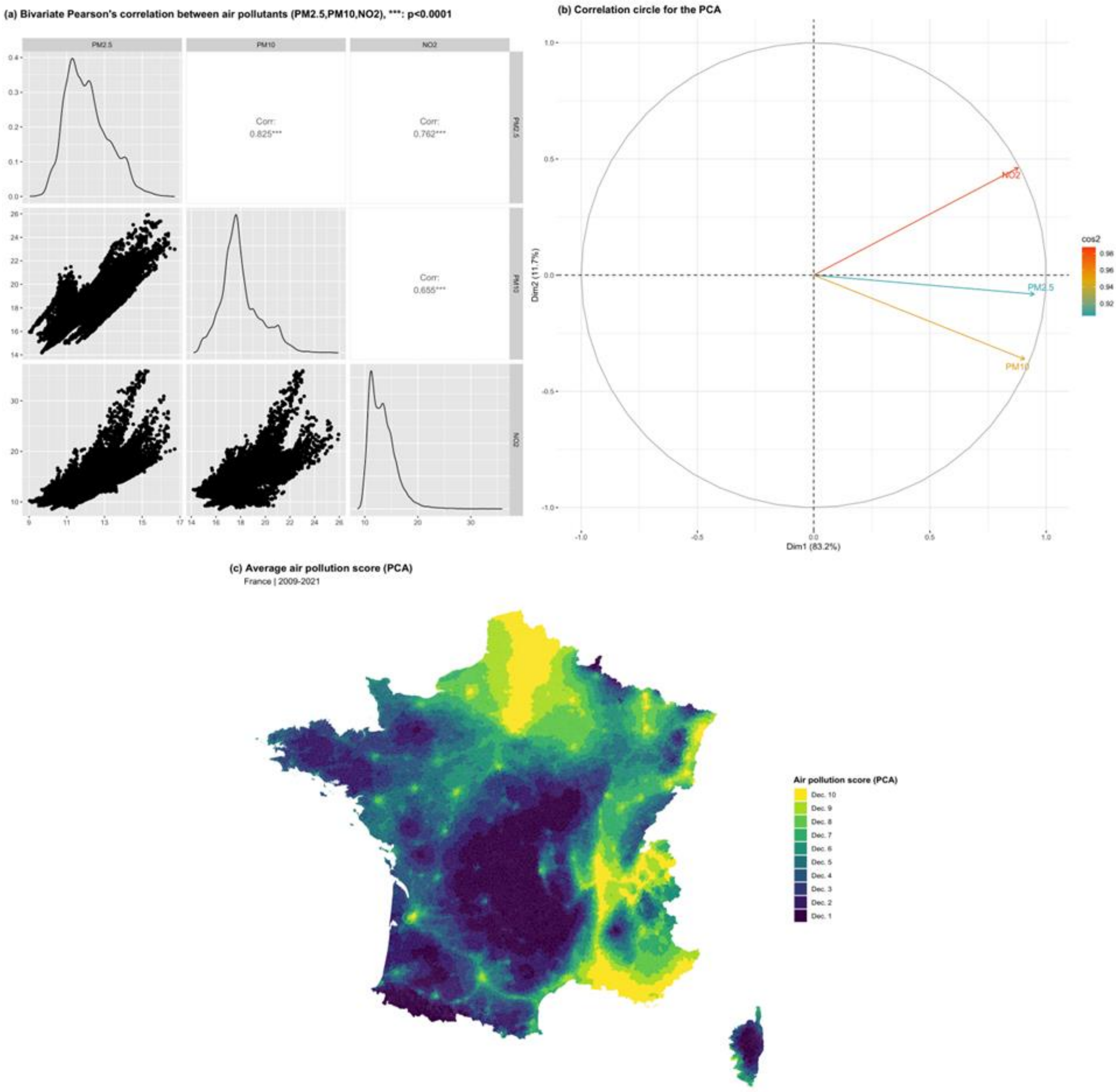

Figure S2. Construction of the air pollution PCA score: (a) bivariate Pearson's correlation between air pollutants ($PM_{2.5}$, PM_{10}, NO_2), (b) correlation circle from the principal component analysis.

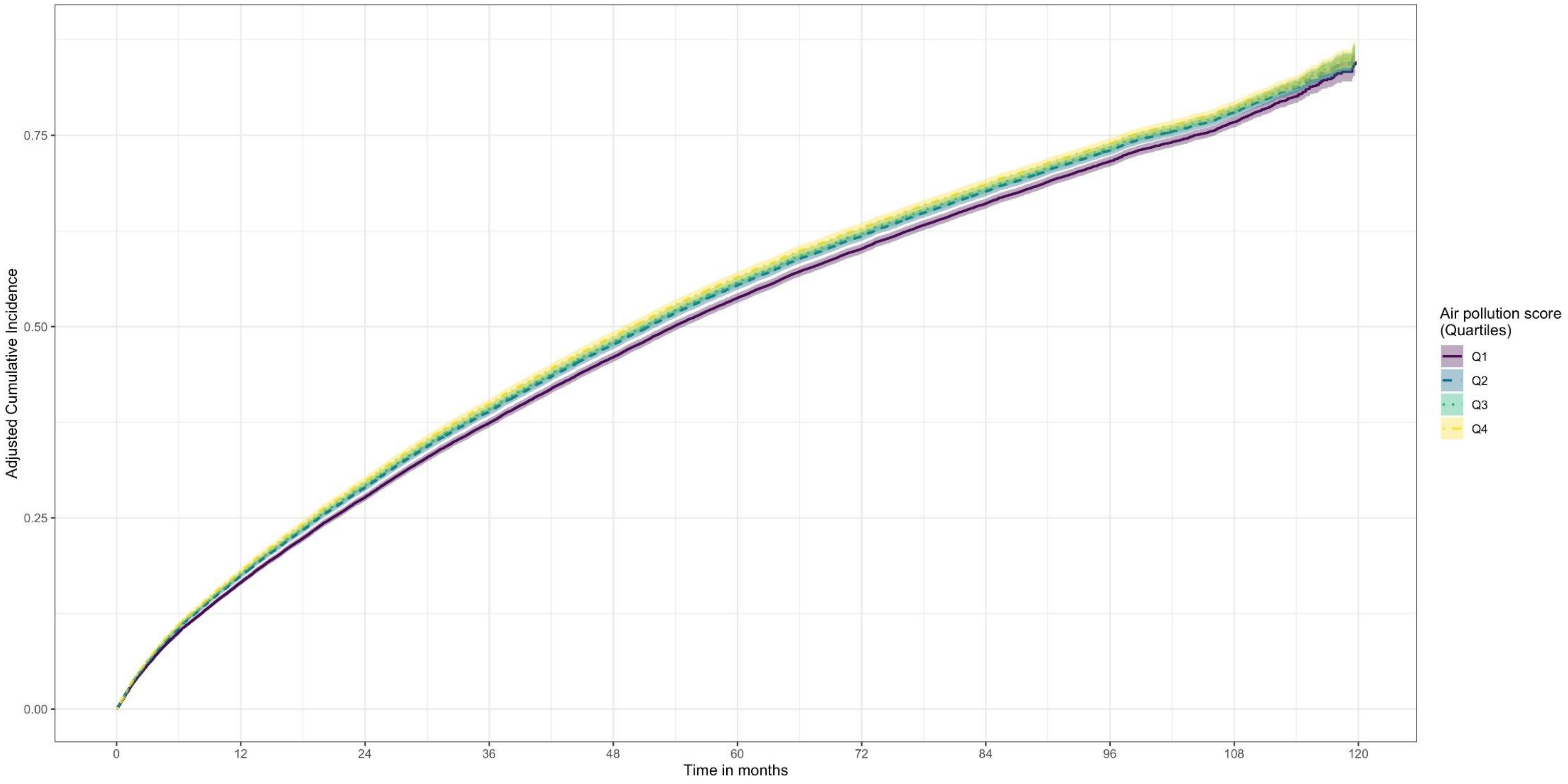

Figure S3. Adjusted cumulative incidence of all-cause mortality according to the air pollution PCA score (quartiles).

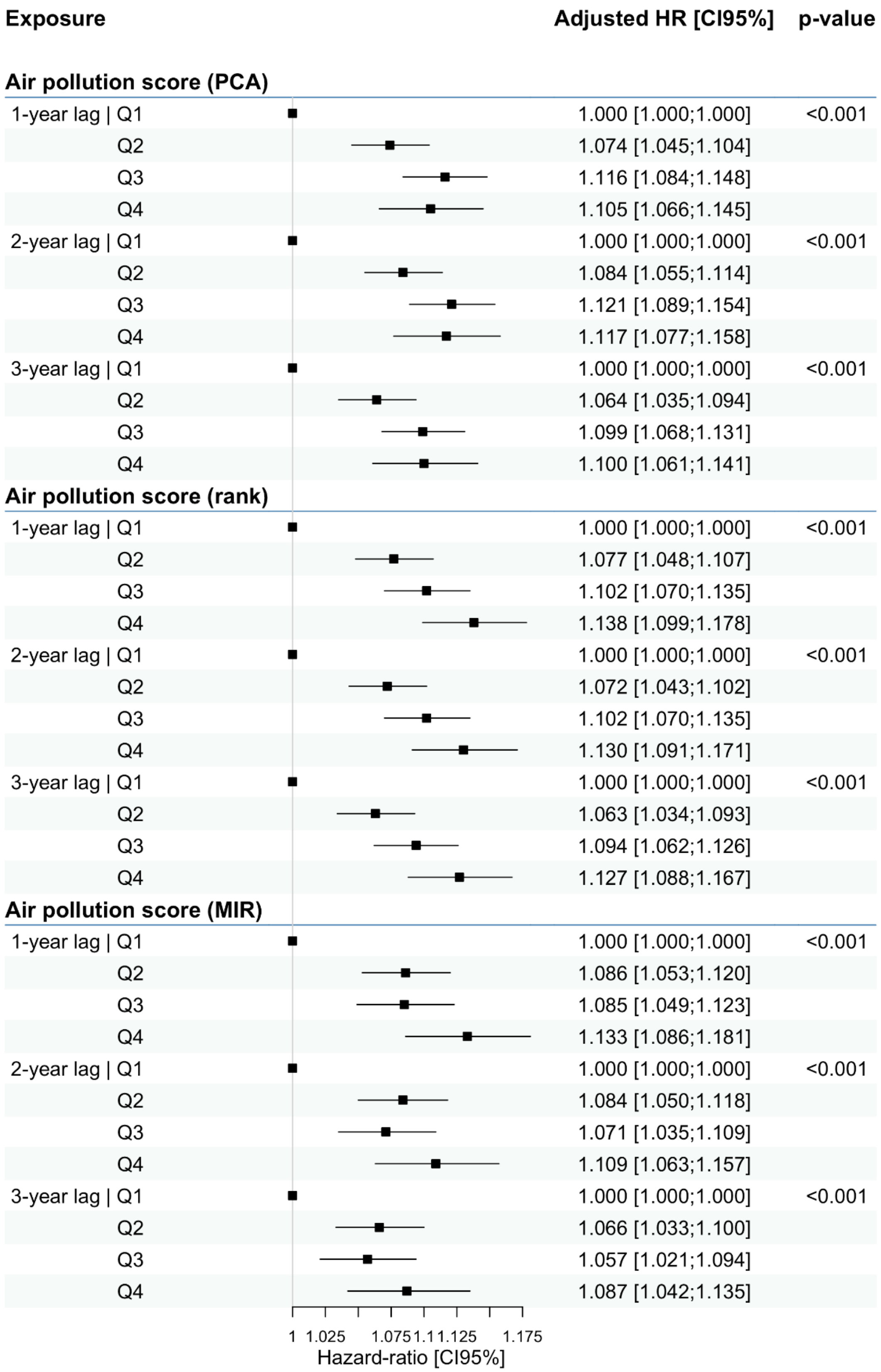

Figure S4. Sensitivity analysis: association between multiple air pollutant exposure and risk of all-cause mortality, according to the alternative composite air pollution scores.

Exposure	Adjusted HR [CI95%]	p-value
**Air pollution score (PCA)**		
1-year lag | Q1	1.000 [1.000;1.000]	<0.001
Q2	1.161 [1.093;1.232]	
Q3	1.130 [1.060;1.206]	
Q4	1.142 [1.053;1.238]	
2-year lag | Q1	1.000 [1.000;1.000]	<0.001
Q2	1.165 [1.097;1.237]	
Q3	1.113 [1.043;1.188]	
Q4	1.147 [1.056;1.245]	
3-year lag | Q1	1.000 [1.000;1.000]	<0.001
Q2	1.156 [1.088;1.228]	
Q3	1.097 [1.028;1.171]	
Q4	1.134 [1.044;1.232]	
**Air pollution score (rank)**		
1-year lag | Q1	1.000 [1.000;1.000]	<0.001
Q2	1.182 [1.114;1.255]	
Q3	1.102 [1.031;1.177]	
Q4	1.182 [1.093;1.277]	
2-year lag | Q1	1.000 [1.000;1.000]	<0.001
Q2	1.157 [1.090;1.229]	
Q3	1.084 [1.015;1.158]	
Q4	1.144 [1.057;1.238]	
3-year lag | Q1	1.000 [1.000;1.000]	<0.001
Q2	1.157 [1.090;1.229]	
Q3	1.069 [1.000;1.141]	
Q4	1.154 [1.066;1.249]	
**Air pollution score (MIR)**		
1-year lag | Q1	1.000 [1.000;1.000]	0.014
Q2	1.101 [1.027;1.180]	
Q3	1.080 [1.000;1.166]	
Q4	1.155 [1.050;1.271]	
2-year lag | Q1	1.000 [1.000;1.000]	0.074
Q2	1.066 [0.995;1.143]	
Q3	1.043 [0.965;1.127]	
Q4	1.118 [1.016;1.230]	
3-year lag | Q1	1.000 [1.000;1.000]	0.589
Q2	1.009 [0.941;1.082]	
Q3	1.016 [0.939;1.098]	
Q4	1.058 [0.961;1.164]	

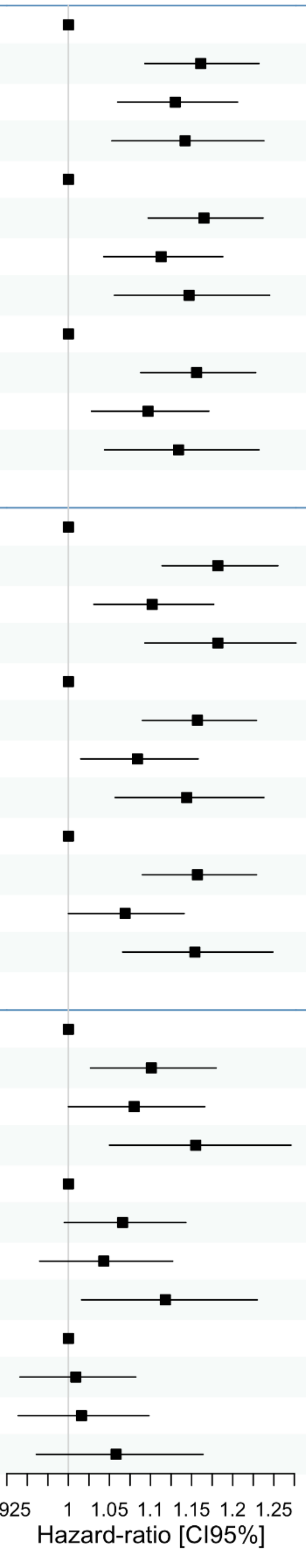

Figure S5. Sensitivity analysis: association between multiple air pollutant exposure and risk of cardiovascular mortality, according to the alternative composite air pollution scores.

Exposure		Adjusted HR [CI95%]	p-value
**Air pollution score (PCA)**			
1-year lag	Q1	1.000 [1.000;1.000]	<0.001
	Q2	1.107 [1.030;1.189]	
	Q3	1.292 [1.199;1.391]	
	Q4	1.329 [1.213;1.457]	
2-year lag	Q1	1.000 [1.000;1.000]	<0.001
	Q2	1.132 [1.052;1.217]	
	Q3	1.299 [1.206;1.399]	
	Q4	1.326 [1.208;1.456]	
3-year lag	Q1	1.000 [1.000;1.000]	<0.001
	Q2	1.064 [0.989;1.144]	
	Q3	1.257 [1.167;1.354]	
	Q4	1.275 [1.161;1.400]	
**Air pollution score (rank)**			
1-year lag	Q1	1.000 [1.000;1.000]	<0.001
	Q2	1.147 [1.067;1.233]	
	Q3	1.270 [1.177;1.370]	
	Q4	1.449 [1.328;1.582]	
2-year lag	Q1	1.000 [1.000;1.000]	<0.001
	Q2	1.123 [1.044;1.207]	
	Q3	1.239 [1.149;1.336]	
	Q4	1.351 [1.236;1.478]	
3-year lag	Q1	1.000 [1.000;1.000]	<0.001
	Q2	1.085 [1.009;1.167]	
	Q3	1.229 [1.140;1.326]	
	Q4	1.367 [1.250;1.495]	
**Air pollution score (MIR)**			
1-year lag	Q1	1.000 [1.000;1.000]	<0.001
	Q2	1.281 [1.184;1.385]	
	Q3	1.304 [1.196;1.421]	
	Q4	1.436 [1.289;1.600]	
2-year lag	Q1	1.000 [1.000;1.000]	<0.001
	Q2	1.318 [1.219;1.425]	
	Q3	1.291 [1.183;1.409]	
	Q4	1.422 [1.275;1.586]	
3-year lag	Q1	1.000 [1.000;1.000]	<0.001
	Q2	1.315 [1.216;1.422]	
	Q3	1.269 [1.161;1.386]	
	Q4	1.417 [1.269;1.581]	

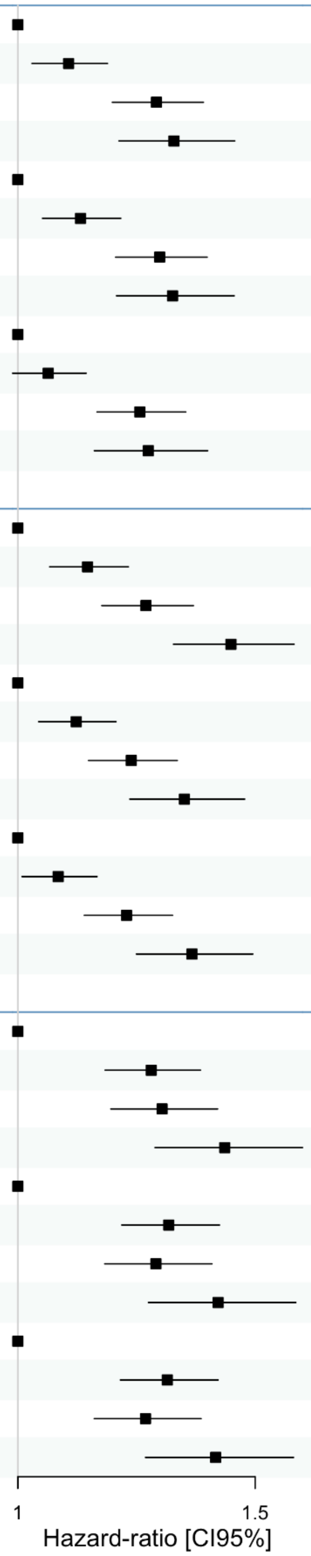

Figure S6. Sensitivity analysis: association between multiple air pollutant exposure and risk of infection-related mortality, according to the alternative composite air pollution scores.
